# Genomic and Phenotypic Differentiation of Ardi Goat Lines with Distinct Facial Pigmentation in Bahrain: Implications for Conservation

**DOI:** 10.3390/ani16121861

**Published:** 2026-06-16

**Authors:** Khaleel Jawasreh, Alessandra Stella, Muneer Al-Najar, Atia Mahmoud, Ebrahim Yusuf, Paul Boettcher, Markos Tibbo

**Affiliations:** 1 Department of Animal Production, Faculty of Agriculture, Jordan University of Science and Technology, Irbid 22110, Jordan; kijawasreh@just.edu.jo; 2 Istituto di Biologia e Biotecnologia Agraria, Consiglio Nazionale delle Ricerche (CNR), 20133 Milan, Italy; alessandra.stella@ibba.cnr.it; 3 Ministry of Municipal Affairs and Agriculture, Manama P.O. Box 251, Bahrain; maebrahim@mun.gov.bh (M.A.-N.); attia.samir2001@yahoo.com (A.M.); eyahmed@mun.gov.bh (E.Y.); 4 Animal Production and Health Division, Food and Agriculture Organization of the United Nations, 00153 Rome, Italy; paul.boettcher@fao.org; 5 Subregional Office for the Gulf Cooperation Council States and Yemen, Food and Agriculture Organization of the United Nations, Al Qala-id Street, Abu Dhabi P.O. Box 62072, United Arab Emirates

**Keywords:** candidate genomic regions, conservation genetics, genome-wide association study, indigenous goats, phenotypic characterization

## Abstract

Indigenous goat populations are valuable genetic resources for livestock production in arid and semi-arid environments, yet many remain insufficiently characterized at the phenotypic and genomic levels. This study investigated phenotypic variation and genome-wide associations in two local Ardi goat lines in Bahrain: Ardi Bahraini and Ardi Mu’atar, the latter being distinguished by a characteristic facial marking pattern. A total of 280 goats were phenotypically characterized for qualitative traits and body measurements, and 76 animals were genotyped using the Illumina Caprine 60K single nucleotide polymorphism (SNP) BeadChip. After quality control, 49,716 autosomal SNPs were retained for genome-wide association analysis. Phenotypic analysis showed that the two lines differed significantly in body weight, body length, hip height, face width, tail length, ear width, and tail circumference, while discriminant analysis identified tail length, ear width, tail circumference, and facial patterns differentiating the lines. Principal component analysis (PCA) showed partial genomic clustering of the two lines, and genome-wide significant and suggestive SNPs based on Bonferroni and false discovery rate (FDR) thresholds on chromosomes 6, 13, 14, and 29. The strongest association was observed for rs268277393 on chromosome 13, located near *DOK5* (Docking Protein 5) and *TRNAC-GCA* (transfer RNA cysteine, anticodon GCA), and was associated with the Ardi Mu’atar facial pattern. Additional candidate regions were located near genes with possible roles in pigmentation, development, or morphological variation. These findings provide preliminary genomic evidence supporting the phenotypic distinctiveness of Ardi Mu’atar goats and identify candidate markers that may contribute to future conservation and breeding programs. Further validation in larger populations and functional studies will be required to confirm the biological role of these candidate regions.

## Simple Summary

Indigenous livestock breeds are valuable genetic resources because they often possess traits that help them survive and produce under local environmental conditions. In Bahrain, the Ardi goat is an important local goat type, and farmers recognize a distinctive line known as Ardi Mu’atar, which is characterized mainly by a unique facial marking pattern. This study compared Ardi Bahraini and Ardi Mu’atar goats using body measurements, visible physical traits, and genome-wide single nucleotide polymorphism (SNP) arrays. The results showed that the two lines differ in several phenotypic traits, particularly facial pattern, tail length, ear width, and tail circumference. Genome-wide association studies (GWASs) identified candidate genomic regions that may be associated with these differences, especially the facial marking pattern of Ardi Mu’atar goats. These findings provide useful preliminary information for the conservation, breeding, and genetic management of local goat resources in Bahrain. Further validation in larger populations will help confirm the markers identified in this study.

## 1. Introduction

Indigenous livestock populations represent important reservoirs of genetic diversity for sustainable animal production, particularly in marginal environments where locally adapted breeds often outperform cosmopolitan breeds under low-input and climatically stressful conditions. Goats are especially important in arid and semi-arid regions because of their ability to utilize sparse vegetation, tolerate heat and water scarcity, and contribute to household income, food security, and cultural identity. In the Arabian Gulf, local goat populations have been shaped by environmental adaptation, traditional husbandry systems, and breeder preferences for specific morphological and productive traits. However, many of these populations remain insufficiently characterized, limiting the development of evidence-based breeding, conservation, and genetic management programs.

Phenotypic characterization is a fundamental first step in documenting animal genetic resources. Morphological traits such as body size, ear dimensions, horn characteristics, coat color, facial pigmentation, and tail morphology can provide useful indicators of population differentiation, farmer selection, and adaptation to local production environments. Standardized phenotypic characterization approaches have been widely recommended for describing livestock populations and supporting conservation planning [1]. Previous studies on indigenous goat populations have shown that body measurements and qualitative traits can distinguish local breeds or ecotypes and reveal patterns of selection, adaptation, or genetic divergence [2,3,4]. In small ruminants, such traits are not only biologically informative but may also carry cultural and economic value, particularly when farmers select animals for visually distinctive phenotypes.

Coat color and pigmentation traits are among the most visible and widely selected characteristics in domestic animals. These traits are influenced by melanin synthesis, distribution, and deposition, including variation in eumelanin and pheomelanin production. More than 150 genes have been associated with pigmentation and color variation in mammals, many of which act through melanocyte development, melanin synthesis, pigment transport, and spatial patterning during development [5,6,7,8]. Classical pigmentation genes such as *MC1R* (Melanocortin 1 Receptor), *ASIP* (Agouti Signaling Protein), and *TYRP1* (Tyrosinase-Related Protein 1) have been linked to coat-color variation in several domestic species, while genome-wide studies continue to identify additional loci and regulatory regions contributing to pigmentation diversity [6,9,10]. Recent genomic studies have demonstrated that pigmentation traits in goats are influenced by multiple loci and structural variants, highlighting their complex genetic architecture [11,12,13]. Distinct pigmentation traits have been documented in several livestock breeds, such as the color-sided patterns in goats and cattle, the black-headed Persian sheep, and the belted phenotype observed in pigs and cattle. Genome-wide association studies (GWAS) in goats have also identified loci associated with coat color, skin pigmentation, and stripe patterns, as reported in Chinese and Mongolian goat populations [14,15]. Although facial pigmentation is primarily a visually selected trait, it may also be associated with breed identity, market preference, and, in some cases, adaptation to environmental conditions such as solar radiation.

The Ardi goat is an important local goat type distributed across the Gulf region. In Bahrain, Ardi goats are valued for their adaptation to hot and humid conditions and their ability to produce under traditional and semi-intensive systems. Within the Bahraini Ardi population, farmers recognize a distinctive line known as Ardi Mu’atar, characterized primarily by a specific lined or striped facial pigmentation pattern. This line appears to have emerged through farmer-driven selection based on esthetic and breed-identity preferences. The more common Ardi Bahraini line and the visually distinctive Ardi Mu’atar line therefore provide a useful model for investigating phenotypic differentiation and the possible genomic basis of a locally selected pigmentation phenotype.

Recent advances in genomic technologies provide new opportunities to characterize indigenous livestock populations beyond observable morphology. Single nucleotide polymorphism arrays and genome-wide association studies can identify genomic regions associated with phenotypic variation and provide candidate markers for breed differentiation, conservation, and selection. In goats, genome-wide approaches have been applied to investigate coat color, mohair traits, adaptation, production traits, and population structure [16,17]. Such tools are particularly valuable for small or locally important populations, where genomic information can support conservation decisions, reduce the risk of genetic dilution, and guide future marker-assisted breeding. Nevertheless, findings from moderate-sized genome-wide association studies should be interpreted cautiously and validated in larger populations before being used for routine selection. Genome-wide association studies have been widely applied in goats to identify candidate loci associated with production, morphological, and pigmentation traits [14,15,18,19]. Indigenous goat populations are also valuable for their adaptation to harsh environments, with genomic studies identifying loci associated with thermoregulation, hypoxia tolerance, and resilience [20,21,22]. Such locally adapted populations represent important reservoirs of genetic diversity for climate-resilient livestock systems [23,24,25].

Although the facial pigmentation pattern is the most visible distinguishing feature, broader phenotypic characterization is necessary to determine whether Ardi Mu’atar represents a distinct line beyond this esthetic trait. This study aimed to (i) characterize phenotypic variation between Ardi Bahraini and Ardi Mu’atar goats, with particular emphasis on the distinctive facial pigmentation pattern, and (ii) identify genomic regions associated with this defining trait using a genome-wide association approach. By integrating phenotypic and genomic analyses, the study provides insight into both general line differentiation and the genetic basis of the facial pigmentation pattern recognized by farmers.

## 2. Materials and Methods

### 2.1. Ethical Approval

This study was conducted under the collaboration agreement between the Food and Agriculture Organization of the United Nations (FAO) and the Ministry of Municipalities Affairs and Agriculture of Bahrain through project. Ethical approval was obtained under protocol number UTF/BAH/006/BAH. Ethical clearance and permission to collect animal phenotypic data and biological samples were obtained through the relevant national and institutional arrangements established for the project. All animal handling and sample collection procedures were performed by trained personnel following standard animal welfare and biosafety practices.

### 2.2. Study Area and Animals

The study was conducted in the Kingdom of Bahrain, covering goat flocks distributed across the four governorates: Capital, Muharraq, Northern, and Southern. Two local Ardi goat lines were included: Ardi Bahraini, the more common local Ardi line, and Ardi Mu’atar, a farmer-recognized line distinguished mainly by its characteristic lined facial pigmentation pattern.

A total of 280 goats with complete phenotypic records were included in the analysis, comprising 231 Ardi Bahraini and 49 Ardi Mu’atar goats. The sampling approach was informed by consultations with livestock experts and local knowledge of Ardi goat distribution in Bahrain. Animals included in the study were considered unrelated based on owner information and field assessment where available.

A subset of 76 unrelated female goats was selected for genomic analysis, comprising 40 Ardi Bahraini and 36 Ardi Mu’atar animals. These animals were selected to represent the two phenotypically distinct lines, with particular attention to the presence or absence of the characteristic Ardi Mu’atar facial pigmentation pattern.

Blood samples were collected by trained technicians using Flinders Technology Associates (FTA) cards (Whatman, GE Healthcare, Chicago, IL, USA). Genomic deoxyribonucleic acid (DNA) was extracted from the FTA-card blood samples using the QIAamp DNA Investigator Kit (Qiagen, Hilden, Germany) according to the manufacturer’s protocol. Extracted DNA samples were assessed for suitability prior to genotyping. Only female animals were selected for genotyping to reduce potential sex-related variation and to ensure a more uniform sampling framework across flocks.

### 2.3. Phenotypic Characterization

Phenotypic data were collected using a standardized questionnaire developed according to FAO guidelines for phenotypic characterization of animal genetic resources [1]. Prior to data collection, the field team received training on the recording of qualitative traits and the measurement of quantitative body traits in small ruminants. The questionnaire and measurement procedures were reviewed and validated before field implementation to ensure consistency and accuracy.

**Figure 1:**
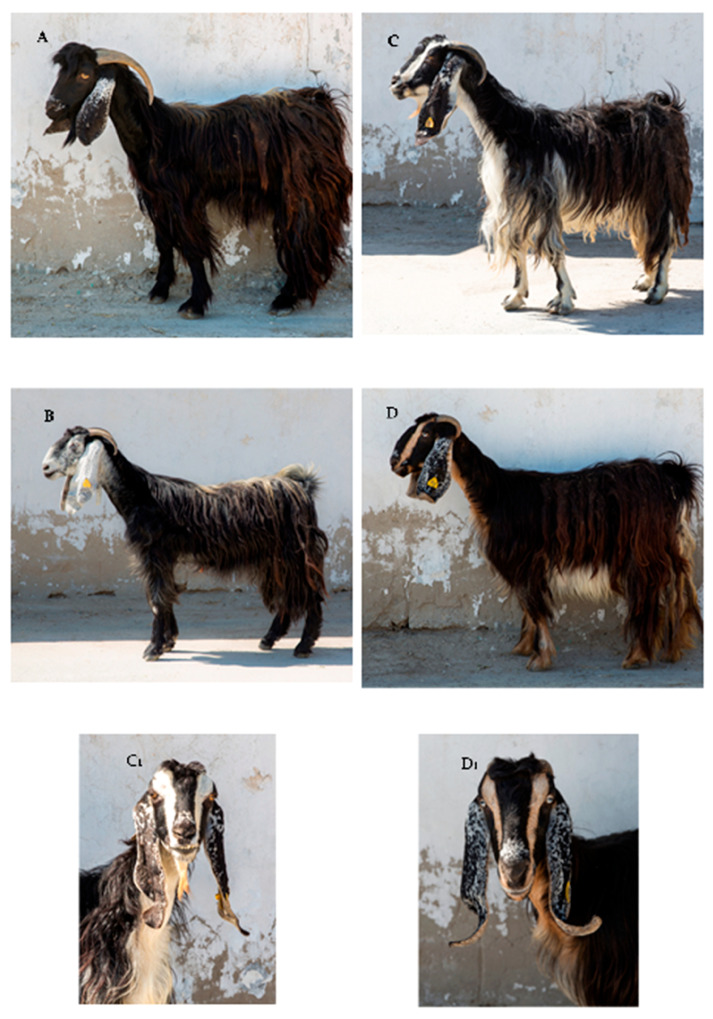
Representative Ardi Bahraini and Ardi Mu’atar goats sampled in Bahrain. (**A**,**B**) Ardi Bahraini goats; (**C**,**D**) Ardi Mu’atar goats showing the characteristic facial pigmentation pattern; (**C_1_**,**D_1_**) close-up facial views of Ardi Mu’atar goats.

Representative animals from the two Ardi goat lines, illustrating the main phenotypic differences in facial pigmentation, are shown in Figure 1. Qualitative traits recorded included body color, facial color and pattern, skin color, body coat pattern, tail type, horn presence and orientation, ear presence and orientation, head profile, hair type, beard presence, and wattles. Particular attention was given to the facial pigmentation pattern because this trait is the primary visible feature used by farmers to distinguish Ardi Mu’atar goats from Ardi Bahraini goats.

Quantitative measurements were recorded in centimeters using standard procedures for small-ruminant phenotypic characterization [1,2,3,4]. Measurements included body length, withers height, hip height, shoulder height, shoulder width, hip width, heart girth/body circumference, face length, face width, ear length, ear width, tail length, tail circumference, horn length, hair length, and testicular circumference where applicable. Live body weight was also recorded for each animal. Additional animal-level information included sex, age group, parity where applicable, line, flock, and geographic location.

### 2.4. Phenotypic Data Management and Statistical Analysis

Field data were entered into a predesigned Microsoft Excel template and checked for completeness, consistency, and possible recording errors. Data screening was performed to identify missing values, implausible values, and potential outliers before statistical analysis. Both male and female goats were included in the phenotypic analyses, except for sex-specific traits (e.g., testicular circumference), which were analyzed only in the relevant subset of animals.

Phenotypic data were analyzed using SAS software version 9.4 (SAS Institute Inc., Cary, NC, USA) [26]. Descriptive statistics for quantitative traits were generated using the PROC MEANS procedure. Frequency distributions for qualitative traits were obtained using the PROC FREQ procedure. Quantitative traits were analyzed using the general linear model procedure, PROC GLM, to evaluate differences between Ardi Bahraini and Ardi Mu’atar goats while accounting for relevant fixed effects and covariates. Similar multivariate and morphometric approaches have been used for phenotypic differentiation of indigenous goat populations in different production systems [2,3,4].

The statistical model used for quantitative traits was:

$$Y_{ijklm}=\mu +L_{i}+S_{j}+A_{k}+R_{l}+bW_{m}+e_{ijklm}$$

where $Y_{ijklm}$ is the observed quantitative trait; μ is the overall mean; $L_{i}$ is the fixed effect of goat line; $S_{j}$ is the fixed effect of sex; $A_{k}$ is the fixed effect of age group; $R_{l}$ is the fixed effect of region/governorate; $W_{m}$ is live body weight was included as a covariate for all linear body measurements where biologically appropriate but was not fitted when body weight itself was the response variable; b is the regression coefficient for body weight; and $e_{ijklm}$ is the residual error.

Line, sex, age group, and region were included as class variables. Least-square means and standard errors were estimated for each line. Differences between means were considered significant at *p* < 0.05, unless otherwise stated.

### 2.5. Canonical Discriminant Analysis and Classification

Canonical discriminant analysis was performed to identify the quantitative traits that contributed most strongly to differentiation between Ardi Bahraini and Ardi Mu’atar goats. The PROC CANDISC and PROC DISCRIM procedures in SAS were used to estimate canonical functions, classification probabilities, and distances between the two lines [13]. Comparable discriminant and multivariate procedures have been applied in previous studies to classify and differentiate indigenous goat populations based on morphological measurements [2,3,4].

Squared Mahalanobis distance was calculated to assess phenotypic divergence between lines using the following expression:

$$D_{ij}^{2}=(x_{i}-x_{j}{)}^{\prime }S^{-1}(x_{i}-x_{j})$$

where $D_{ij}^{2}$ is the squared Mahalanobis distance between lines i and j, $x_{i}$ and $x_{j}$ are the vectors of trait means for the two lines, and $S^{-1}$ is the inverse of the pooled covariance matrix. Traits with the highest discriminatory power were considered key phenotypic descriptors separating the two Ardi goat lines.

### 2.6. SNP Genotyping and Quality Control

Genotyping was performed using the Illumina Caprine 60K SNP BeadChip (Illumina Inc., San Diego, CA, USA), which contains approximately 60,000 single nucleotide polymorphism markers distributed across the goat genome. SNP-array platforms have been widely used for genome-wide association and population-genomic studies in goats and other domestic animals [27,28]. GWAS has been widely used in goats to identify associations between genomic variation and complex traits [29,30]. High-density SNP arrays and whole-genome approaches provide suitable resolution for identifying candidate regions associated with phenotypic traits [31].

Genotype quality control was conducted using PLINK v1.9 [16]. The initial dataset included 54,485 autosomal SNPs. The following filtering criteria were applied: SNPs and individuals with a genotype call rate below 90% were excluded; SNPs with a minor allele frequency (MAF) below 0.05 were removed to avoid low-frequency variants; and SNPs showing significant deviation from Hardy–Weinberg equilibrium (HWE; *p* < 0.0001) were excluded. After quality control, 76 animals and 49,716 high-quality autosomal SNPs were retained for downstream genome-wide association analysis.

### 2.7. Genome-Wide Association Analysis

Genome-wide association analysis was performed to identify SNPs associated with phenotypic differentiation between Ardi Bahraini and Ardi Mu’atar goats. The primary phenotype analyzed was the line-defining facial pigmentation pattern characteristic of Ardi Mu’atar goats. This phenotype was defined as a binary variable, coded as 1 for animals exhibiting the lined/striped facial pattern (Ardi Mu’atar) and 0 for animals lacking this pattern (Ardi Bahraini). Although line identity and facial pattern are closely aligned in this population, the GWAS was conducted explicitly using the presence/absence of the facial stripe phenotype as the response variable.

Because the facial pigmentation phenotype is strongly aligned with farmer-defined line identity, the GWAS design inherently involves a degree of confounding between phenotype and population structure. Therefore, the analysis was interpreted cautiously, and the identified associations were considered as candidate genomic regions potentially reflecting both pigmentation-related loci and underlying genomic differentiation between the two lines. Input files were prepared using R version 4.2.2 (R Core Team, Vienna, Austria) and PLINK version 1.9 [27,28], and association analysis was performed using RTM-GWAS software version 1.0 (Huazhong Agricultural University, Wuhan, China) [32].

The primary association phenotype was the line-defining facial pigmentation pattern, coded according to the presence or absence of the Ardi Mu’atar facial line phenotype. Association results were evaluated using SNP-level *p*-values. Multiple-testing correction was performed using both the Bonferroni correction and the false discovery rate (FDR) procedure implemented in R [28]. Genome-wide significance thresholds were set using Bonferroni correction (*p* < 1 × 10^−5^) and FDR-adjusted thresholds (q < 0.05). Manhattan and Q–Q plots were generated using the qqman package in R. Genomic inflation was evaluated using Manhattan plots and quantile–quantile plots; however, genomic inflation factors (λ) were not formally estimated and should be considered in future analyses with larger datasets. A mixed-model GWAS approach incorporating a genomic relationship matrix was not applied in this study because of the modest sample size and the exploratory nature of the analysis. Instead, the results were interpreted as preliminary and hypothesis-generating.

Because the genomic dataset represented two phenotypically defined local lines with a modest sample size, the GWAS results were interpreted as exploratory and were used to identify candidate genomic regions for further validation rather than definitive causal variants. Similar caution has been recommended in livestock genome-wide association studies where sample size, population structure, and relatedness may influence marker-trait associations [32].

**Figure 2:**
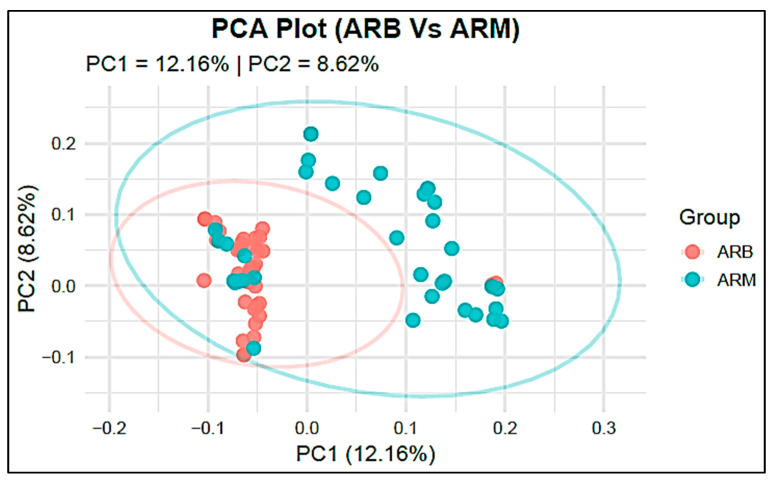
Principal component analysis (PCA) of Ardi Bahraini (ARB) and Ardi Mu’atar (ARM) goats based on filtered SNP genotyping data. Points are color-coded by line to facilitate visualization of clustering patterns. The first two principal components explained 12.16% and 8.62% of the total genomic variation, respectively. The plot shows partial genetic clustering with some overlap, indicating moderate genomic differentiation and shared ancestry. Note: Ellipses represent the 95% confidence intervals for each group, indicating the dispersion and clustering of individuals within the Ardi Bahraini and Ardi Mu’atar populations based on principal component scores.

To assess genomic clustering and potential line structure between Ardi Bahraini and Ardi Mu’atar goats, principal component analysis (PCA) was performed using PLINK v1.9 based on the filtered SNP dataset. The first two principal components were plotted to visualize genomic differentiation between the two lines (Figure 2). Because the facial phenotype was closely aligned with line identity, the GWAS findings were interpreted as exploratory candidate regions rather than causal associations.

### 2.8. Candidate Gene Annotation

Significant and suggestive SNPs identified by the GWAS were mapped to the *Capra hircus* reference genome (ARS1 assembly) using their chromosome and physical positions. Genes located within or near associated SNP regions were identified from available genome annotation resources. Candidate genes were evaluated based on their proximity to associated SNPs and their reported biological functions in pigmentation, development, morphology, or related pathways in goats or other mammalian species.

Because functional validation was not performed in the present study, associated loci were interpreted as candidate genomic regions rather than confirmed causal mutations. Literature-based interpretation was used to discuss possible biological relevance and to identify genomic regions that warrant further validation in larger Bahraini and regional goat populations. Previous studies have shown that genome-wide approaches can identify candidate regions associated with coat color, pigmentation, and other phenotypic traits in goats and other livestock species [7,8,9,10,16,17,27,28].

## 3. Results

### 3.1. Distribution of Sampled Ardi Goat Lines Across Governorates

The phenotypic dataset included Ardi goat populations sampled from the four governorates of Bahrain. Ardi Mu’atar goats were mainly represented in the Northern and Southern governorates, whereas Ardi Bahraini goats were more widely distributed across the sampled areas. Ardi Mu’atar goats accounted for a smaller proportion of the total sampled population, reflecting their more limited occurrence within the surveyed flocks. The geographic distribution of the sampled animals is presented in Table 1.

### 3.2. Phenotypic Differentiation

After adjustment for region, sex, age group, and body weight where appropriate, several quantitative traits differed between Ardi Bahraini and Ardi Mu’atar goats (Table 2). Ardi Bahraini goats had higher least-square mean body weight than Ardi Mu’atar goats, with mean values of 35.20 ± 1.04 kg and 30.83 ± 1.42 kg, respectively. Ardi Bahraini goats also showed greater body length, face width, ear width, tail length, and tail circumference. In contrast, Ardi Mu’atar goats had greater hip height than Ardi Bahraini goats.

No significant differences were observed between the two lines for several other measurements, including shoulder height, ear length, withers height, body circumference, horn length, shoulder width, and testicular circumference. These findings indicate that the two lines are morphologically similar for many body dimensions but differ in a limited set of traits, particularly those related to body size, ear width, tail morphology, and facial appearance.

Qualitative trait assessment showed that both Ardi Bahraini and Ardi Mu’atar goats shared several general external characteristics typical of local Ardi goats. Most animals had a normal body coat pattern, and black was the predominant body color in both lines. Horned animals were more common than polled animals, and backward-curved horns and pendulous ears were frequently observed. Wattles and beards were also present in some animals.

The most distinctive qualitative trait separating the two lines was facial pigmentation. Ardi Mu’atar goats were characterized by a lined or striped facial pattern locally recognized by breeders as a defining feature of the line. This facial pattern was absent in Ardi Bahraini goats, in which black and other facial colors were more common. The presence of this line-specific facial marking supports the field recognition of Ardi Mu’atar as a distinct farmer-selected line within the Bahraini Ardi goat population.

Canonical discriminant analysis identified a small number of traits that contributed most strongly to differentiation between the two Ardi goat lines. The main discriminatory traits were tail length, ear width, tail circumference, and the lined facial pattern characteristic of Ardi Mu’atar goats. The squared Mahalanobis distance between the two lines was 0.318, indicating measurable but relatively modest phenotypic divergence.

The relatively small Mahalanobis distance between Ardi Bahraini and Ardi Mu’atar goats reflects a close phenotypic background, consistent with their origin within the broader Ardi goat population. The discriminant analysis identified a limited set of morphological and pigmentation traits that differentiate the two lines.

### 3.3. Genomic Analyses

A total of 76 unrelated female goats were included in the genomic analysis, comprising 40 Ardi Bahraini and 36 Ardi Mu’atar goats. Genotyping generated an initial dataset of 54,485 autosomal SNPs. After quality control based on genotype call rate and Hardy–Weinberg equilibrium filtering, 49,716 autosomal SNPs were retained for downstream genome-wide association analysis. The retained SNP dataset provided genome-wide marker coverage for identifying genomic regions associated with phenotypic differentiation between the two Ardi goat lines.

Principal component analysis based on the filtered SNP dataset (Figure 2) showed partial clustering of animals according to line, with some overlap between Ardi Bahraini and Ardi Mu’atar goats. The first principal component (PC1) explained 12.16% of the total genomic variation, whereas the second principal component (PC2) explained 8.62%. This PCA plot (Figure 2) displays both separation and overlap between the two groups. The GWAS phenotype (presence or absence of the characteristic facial pigmentation pattern) was closely aligned with line identity, the GWAS results are presented as candidate genomic regions.

**Figure 3:**
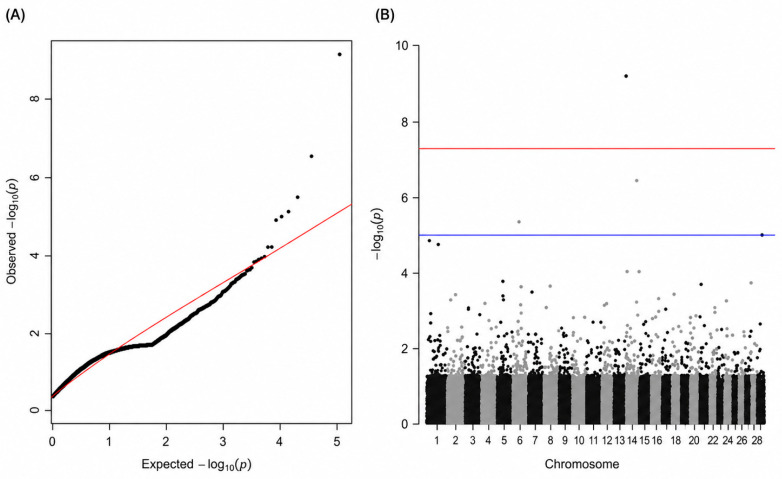
Genome-wide association results for phenotypic differentiation between Ardi Bahraini and Ardi Mu’atar goats using false discovery rate correction. (**A**) Quantile–quantile plot showing the distribution of observed versus expected −log_10_-transformed *p*-values. (**B**) Manhattan plot showing genome-wide SNP associations across autosomes. Note: The blue and red horizontal lines indicate the suggestive and genome-wide significance thresholds, respectively. Each point represents a single nucleotide polymorphism (SNP); grey dots indicate all tested SNPs across the genome, while black dots highlight SNPs exceeding the suggestive or genome-wide significance thresholds. Each point represents an observed versus expected −log_10_(*p*-value); grey dots show the distribution of all SNPs, while black dots emphasize the most significant deviations from the null distribution. The strongest association signal was observed on chromosome 13 near *DOK5* and *TRNAC-GCA*, corresponding to the SNP rs268277393/snp45555-scaffold622-996214.

**Figure 4:**
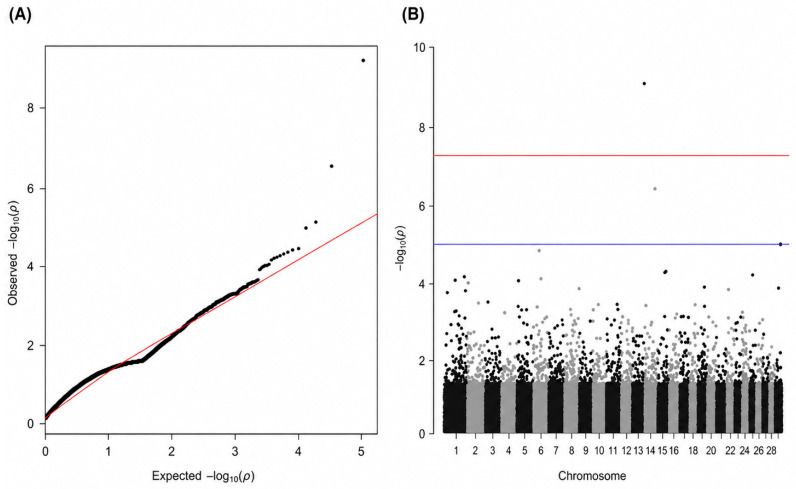
Genome-wide association results for phenotypic differentiation between Ardi Bahraini and Ardi Mu’atar goats using Bonferroni correction. (**A**) Quantile–quantile plot showing the distribution of observed versus expected −log_10_-transformed *p*-values after Bonferroni adjustment. (**B**) Manhattan plot showing SNP associations across autosomes under the Bonferroni correction framework. Note: The blue and red horizontal lines indicate the suggestive and genome-wide significance thresholds, respectively. Each point represents a single nucleotide polymorphism (SNP); grey dots indicate all tested SNPs across the genome, while black dots highlight SNPs exceeding the suggestive or genome-wide significance thresholds. Each point represents an observed versus expected −log_10_(*p*-value); grey dots show the distribution of all SNPs, while black dots emphasize the most significant deviations from the null distribution. The chromosome 13 locus near *DOK5* and *TRNAC-GCA* remained the strongest candidate genomic region associated with the Ardi Mu’atar facial pigmentation phenotype.

Genome-wide association studies identified four SNPs associated with phenotypic differentiation between Ardi Bahraini and Ardi Mu’atar goats, particularly the facial pigmentation phenotype that distinguishes Ardi Mu’atar goats. The associated SNPs were located on chromosomes 6, 13, 14, and 29 (Table 3, Table 4, Table 5 and Table 6; Figure 3 and Figure 4). The binary encoding of the facial pigmentation phenotype differentiated the two line groups used in the analysis.

The strongest association was detected on chromosome 13 at snp45555-scaffold622-996214/rs268277393, located at position 82,031,445 bp. This SNP showed the lowest *p*-value under both FDR and Bonferroni correction (*p* = 6.26 × 10^−10^) and was located near *TRNAC-GCA* and *DOK5*. This locus showed differences in allele and genotype frequencies between the two lines.

A second SNP, snp32665-scaffold3753-271193/rs268264845, was identified on chromosome 14 at position 73,435,116 bp. This SNP was located near *TMEM71* and *PHF20L1* and remained significant after multiple-testing correction (*p* = 3.59 × 10^−7^).

A third SNP, snp41814-scaffold544-72928, was detected on chromosome 6 at position 50,510,087 bp. This marker met the significance thresholds under both FDR and Bonferroni-adjusted analyses. The minor allele frequency differed slightly between the two lines.

A fourth SNP, snp23327-scaffold2330-184378/rs268255751, was identified on chromosome 29 at position 45,098,688 bp. This SNP was located near *LOC102180829* and *SPTBN2*. It met the threshold under the FDR-based analysis but did not remain significant after Bonferroni correction.

Given the modest sample size, the detected associations should be interpreted cautiously as preliminary signals requiring independent validation.

Allele-frequency analysis showed clear differences between the two lines for the chromosome 13 SNP, snp45555-scaffold622-996214. In Ardi Bahraini goats, the A and G alleles were present at equal frequencies, whereas in Ardi Mu’atar goats the G allele was predominant. The GG genotype was observed in 33 of 36 Ardi Mu’atar goats, compared with 11 of 40 Ardi Bahraini goats.

For the chromosome 14 SNP, snp32665-scaffold3753-271193, the minor allele frequency differed between the two lines, with the G allele less frequent in Ardi Mu’atar goats than in Ardi Bahraini goats. The AA genotype was more frequent in Ardi Mu’atar goats, whereas genotype frequencies were more evenly distributed in Ardi Bahraini goats.

For the chromosome 6 SNP, snp41814-scaffold544-72928, allele frequencies were similar between the two lines.

For the chromosome 29 SNP, snp23327-scaffold2330-184378, the G allele was more frequent in Ardi Mu’atar goats than in Ardi Bahraini goats, and heterozygous animals were common in the Ardi Mu’atar group. This SNP met the threshold under the FDR-based analysis but did not remain significant after Bonferroni correction.

### 3.4. Candidate Genomic Regions

The associated SNPs identified in this study were located near genes related to pigmentation, development, or morphological variation. The region on chromosome 13 included rs268277393, which was located near *DOK5* and *TRNAC-GCA*. This SNP showed the lowest *p*-value and differences in allele frequencies between the two lines.

Additional regions were identified on chromosomes 14 and 29, near *TMEM71*, *PHF20L1*, *LOC102180829*, and *SPTBN2*. These loci are presented as candidate genomic regions associated with the phenotype.

## 4. Discussion

### 4.1. Phenotypic Differentiation

This study provides evidence of phenotypic and genomic differentiation between Ardi Bahraini and Ardi Mu’atar goats, two locally recognized Ardi goat lines in Bahrain. Although the two lines share a common phenotypic background as part of the broader Ardi goat population, several traits differentiated them, particularly facial pigmentation pattern, tail length, ear width, and tail circumference. Ardi Mu’atar goats were most clearly distinguished by the lined facial pattern recognized by local breeders, whereas Ardi Bahraini goats had higher mean values for several body measurements, including body weight, body length, ear width, face width, tail length, and tail circumference. These findings indicate that the Ardi Mu’atar line has a recognizable phenotype that may reflect farmer-driven selection for a distinctive facial marking pattern within the local Ardi goat population. This interpretation was further supported by PCA (Figure 2), which showed partial genomic separation between the two lines, although the observed overlap indicates that Ardi Bahraini and Ardi Mu’atar remain closely related populations.

Phenotypic characterization remains an essential first step in the documentation and management of animal genetic resources, especially in local populations for which pedigree and genomic information are limited [1]. Body measurements and qualitative traits have been widely used to describe and differentiate indigenous goat populations under traditional production systems [2,3,4]. Similar to previous studies on Ethiopian, Rwandan, and West African dwarf goats, the present findings show that a limited number of morphometric and visible traits can provide useful descriptors for distinguishing closely related goat populations [2,3,4]. The relatively small Mahalanobis distance observed between Ardi Bahraini and Ardi Mu’atar goats suggests that the two lines are not deeply divergent at the phenotypic level but have differentiated in specific traits of breeder and cultural relevance.

The observed differences in body weight and body dimensions may reflect a combination of genetic background, breeder preference, flock management, sex and age structure, and environmental effects. Although the statistical model adjusted for major fixed effects, phenotypic traits in small ruminants are influenced by nutrition, management system, parity, health status, and production purpose. Therefore, body-size differences between the two lines should be interpreted cautiously and validated in larger, more balanced datasets. In contrast, the facial line pattern of Ardi Mu’atar appears to be a stable and visually distinctive trait that is central to the local recognition of this line.

Coat color and facial pigmentation are among the most visible traits used by farmers and breeders to identify and select animals. In this study, the lined facial pigmentation pattern was the principal qualitative trait distinguishing Ardi Mu’atar goats from Ardi Bahraini goats. Such traits may not always have direct production value, but they often carry important cultural, esthetic, market, and breed-identity significance. In many livestock systems, farmer preferences for color pattern, horn shape, body conformation, or other visible traits contribute to the formation of local lines or ecotypes.

Pigmentation traits in mammals are controlled by complex biological pathways involving melanocyte development, melanin synthesis, pigment distribution, and spatial patterning during development [5,6,7,8]. Variation in eumelanin and pheomelanin production contributes to differences in black, brown, red, and lighter pigmentation patterns, while spatial regulation of melanocyte migration and pigment deposition contributes to markings and patterned phenotypes [5,6]. Classical genes such as *MC1R*, *ASIP*, and *TYRP1* have been associated with coat-color variation in several domestic species [9,10], but genome-wide studies increasingly show that pigmentation phenotypes may involve multiple loci, regulatory regions, and developmental pathways [6,7,8,16]. Consistent with this study, GWAS and genomic analyses in goats have identified multiple loci influencing coat color and pigmentation patterns, often involving complex genetic mechanisms [11,14].

The distinctive facial marking of Ardi Mu’atar goats may therefore reflect selection on one or more loci affecting pigment distribution in the facial region. However, because the phenotype was investigated in a modest genomic sample and functional validation was not performed, the genetic signals identified in this study should be interpreted as candidate regions rather than confirmed causal mutations.

### 4.2. Genomic Insights and Pigmentation

The genome-wide association analysis identified four SNPs on chromosomes 6, 13, 14, and 29 associated with phenotypic differentiation between Ardi Bahraini and Ardi Mu’atar goats. The strongest signal was detected on chromosome 13 at rs268277393/snp45555-scaffold622-996214, located near *DOK5* and *TRNAC-GCA*. This marker showed a highly significant association and a marked difference in genotype distribution between the two lines, with the GG genotype being predominant in Ardi Mu’atar goats. This result suggests that the chromosome 13 region may be associated with the line-defining facial pigmentation pattern of Ardi Mu’atar goats.

The identification of candidate genomic regions associated with pigmentation is consistent with previous GWAS in goats, including Markhoz goats [16] and Chinese indigenous breeds [17], which also reported multiple loci influencing coat color and pigmentation traits. These studies highlight the polygenic and population-specific nature of pigmentation phenotypes in goats. The identification of multiple candidate loci is consistent with a previous GWAS in goats, where pigmentation has been shown to be polygenic and population-specific [16,17]. The possible involvement of the chromosome 13 region is consistent with previous goat genomic studies that reported candidate regions for coat-color traits on different chromosomes, including chromosome 13 [16,17]. However, because PCA indicated partial line-level clustering, this association should be interpreted as a candidate signal linked to both facial pigmentation and genomic differentiation between the two lines. Nazari-Ghadikolaei et al. [16] identified candidate genes associated with coat color and mohair traits in Iranian Markhoz goats, demonstrating the value of genome-wide approaches for investigating pigmentation traits in goats. Guo et al. [17] also reported selection signals and genomic regions underlying phenotypic characteristics in Chinese domestic goat breeds. Together, these studies support the broader relevance of genome-wide approaches for identifying candidate regions associated with visible phenotypes in goats.

The proximity of the strongest chromosome 13 SNP to *DOK5* and *TRNAC-GCA* is biologically interesting, although not sufficient to infer causality. *DOK5* has been discussed in relation to pigmentation-related pathways in other animal studies, while transfer RNA genes may influence protein synthesis processes relevant to hair and skin biology. Cysteine availability and sulfur-containing amino acid metabolism are important for hair structure because hair keratins are rich in cysteine residues, and cysteine has been associated with hair growth and fiber characteristics [33,34]. Nevertheless, the functional connection between this specific SNP, nearby genes, and the Ardi Mu’atar facial pattern remains hypothetical and requires further investigation. Previous genome-wide studies have similarly identified multiple candidate regions associated with phenotypic traits in goats, reflecting the polygenic nature of these traits [19,29]. Structural variants have also been implicated in pigmentation variation in goats, further highlighting the complexity of the genetic architecture [11,31].

The chromosome 14 SNP located near *TMEM71* and *PHF20L1* also remained significant after multiple-testing correction. Although the direct role of these genes in goat pigmentation is not established, they may be involved in developmental or regulatory pathways. The chromosome 29 region near *LOC102180829* and *SPTBN2* showed suggestive evidence of association but did not remain significant after Bonferroni correction. *SPTBN2* has been associated mainly with neurological and developmental phenotypes in other species [35,36,37], and its direct relationship with pigmentation in goats remains uncertain. Therefore, this locus should be regarded as suggestive and requiring further validation.

The chromosome 6 SNP showed statistical evidence of association, but the difference in allele frequency between the two lines was relatively small. This may indicate a weaker effect, linkage with another causal variant, or a signal influenced by the structure of the sampled population. Overall, the chromosome 13 region appears to be the strongest and most biologically relevant candidate region identified in this study.

### 4.3. Conservation Implications and Limitations

Genomic studies have demonstrated that goats possess extensive adaptive genetic variation, including loci associated with environmental stress tolerance and production efficiency [20,22]. The results support the recognition of Ardi Mu’atar as a phenotypically distinct line based primarily on facial pigmentation. Conservation strategies should therefore focus on preserving this trait while maintaining overall genetic diversity [1].

The Ardi goat is adapted to the hot and humid conditions of the Gulf region and maintaining diversity within local goat populations is important for future breeding and adaptation. The Ardi Mu’atar line may have cultural and market value because of its distinctive facial marking. If farmers continue to select for this phenotype, genomic information could eventually help support controlled mating, conservation of the line, and avoidance of unplanned dilution through crossbreeding. However, the markers identified in this study should not yet be used as definitive selection tools. They require validation in larger and independent populations before being incorporated into marker-assisted selection or conservation breeding programs.

A balanced conservation strategy should preserve the unique Ardi Mu’atar phenotype while maintaining sufficient genetic diversity. Selection based only on a narrow visual trait could increase inbreeding or reduce genetic variation if not managed carefully. Therefore, any future breeding program should combine farmer preferences, phenotypic characterization, genomic diversity assessment, and population management principles. Additional analyses such as runs of homozygosity, effective population size, genomic inbreeding, admixture analysis, and within-line diversity estimates would strengthen conservation recommendations. Preserving such locally adapted genetic resources is increasingly important under climate change scenarios [23,24,25].

This study offers initial genomic indications of differentiation between Ardi Bahraini and Ardi Mu’atar goats; however, several limitations should be considered. Notably, the genomic sample size was relatively small, with only 76 animals genotyped. Although adequate to identify strong differentiation at certain loci, a limited sample size may reduce statistical power, increase the likelihood of false positives or negatives, and constrain the detection of loci with minor effects. Consequently, the GWAS results should be viewed as exploratory.

Second, the phenotype analyzed in the GWAS was closely related to line identity. Because the GWAS phenotype (presence/absence of facial stripes) was closely aligned with farmer-defined line identity, the association signals may reflect both pigmentation-related variation and broader genomic differentiation between the two lines. Population structure and relatedness can confound association studies if not adequately controlled. In this study, PCA revealed partial clustering of the two goat lines, indicating moderate genomic differentiation while also suggesting that the underlying line structure may influence association signals. An important methodological consideration is the strong correspondence between the GWAS phenotype (presence/absence of facial stripes) and line identity, which introduces potential confounding between true phenotype-associated loci and signals driven by population structure. Although the PCA results indicate only moderate genomic separation, some associations—particularly the strong signal on chromosome 13—may reflect both pigmentation effects and broader line differentiation. The genomic sample size (n = 76) is relatively small for GWAS analyses and limits statistical power. While strong signals such as the chromosome 13 locus were detected, associations with smaller effects may have gone undetected, and some observed signals may represent false positives. Therefore, the reported associations should be considered preliminary and require validation in larger and independent populations. Genomic inflation factors were not formally calculated in this study, which limits the ability to fully assess residual population structure effects. Mixed-model GWAS approaches that account for genomic relationships were not implemented, which represents a limitation. Such approaches would better control for population structure and should be a priority in future studies with larger sample sizes. Future analyses using larger datasets should incorporate genomic relationship matrices, mixed-model GWAS, genomic inflation estimates, and independent validation populations to better distinguish true marker–phenotype associations from effects related to population structure.

Third, the candidate genes were identified based on physical proximity to associated SNPs and literature-based functional interpretation. This approach is useful for generating hypotheses but does not prove biological causality. Functional validation, gene-expression analysis in skin or hair follicles, fine mapping, and whole-genome sequencing would be needed to identify causal variants and clarify the mechanisms underlying the facial pigmentation pattern.

Fourth, the study focused on SNP-array data. While SNP chips are useful for genome-wide screening, they may miss rare variants, structural variants, copy-number variants, and regulatory mutations that could contribute to pigmentation patterns. Whole-genome sequencing of representative Ardi Bahraini and Ardi Mu’atar goats would provide higher resolution and may identify causal or more strongly linked variants.

Recent advances in goat genomics, including pangenome analysis and structural variant detection, are expected to improve the identification of causal variants underlying complex traits [30,31]. Minor differences in sample size among phenotypic traits reflected missing measurements for specific variables, and analyses were conducted using the available records for each trait.

Future work should validate the chromosome 13 candidate region in a larger sample of Ardi goats from Bahrain and, where possible, related Ardi populations from other Gulf countries. Such validation should include animals with and without the facial line phenotype, balanced representation of sexes and age groups, and detailed flock-level metadata. Whole-genome sequencing could be used to identify causal variants or regulatory elements linked to facial pigmentation. Gene-expression studies in skin or hair follicles from marked and unmarked facial regions would further clarify whether candidate genes near associated SNPs are involved in pigment deposition.

Additional population-genomic analyses would also be valuable. Estimating genomic diversity, inbreeding, effective population size, admixture, and genetic distance between Ardi Bahraini and Ardi Mu’atar goats would provide a stronger foundation for conservation planning. Integrating genomic data with farmer knowledge, production traits, reproductive performance, and adaptation indicators would allow the development of a more complete conservation and breeding strategy for Bahraini goat genetic resources.

## 5. Conclusions

This study demonstrates that Ardi Mu’atar goats represent a phenotypically recognizable line primarily defined by a distinctive facial pigmentation pattern, with moderate genomic differentiation from the broader Ardi population. Genome-wide analysis identified candidate regions associated with this trait, particularly on chromosome 13, although these findings remain preliminary due to sample size and potential population structure effects. The integration of phenotypic and genomic data provides a useful foundation for conservation planning and highlights the need for further validation studies in larger populations.

## Figures and Tables

**Table 1:** Ardi goat lines/strains and governorates of Bahrain included in the study.

Governorate/ Ardi Line or Strain	Capital		Muharraq		North		South		Total	
	n	(%)	n	(%)	n	(%)	n	(%)	n	(%)
Ardi Mu’atar	0	(0.0)	5	(10.2)	29	(59.2)	15	(30.6)	49	(100)
Ardi Bahraini	12	(5.2)	4	(1.7)	112	(48.1)	105	(45.1)	233	(100)

**Table 2:** Least square means ± SE of phenotypic measurements as affected by line/strain, after adjusting the records for region, weight and sex of goat.

Breed	Ardi Mu’atar	Ardi Bahraini
N	49	232
Body weight (kg)	30.83 ± 1.42 ^b^	35.20 ± 1.04 ^a^
Body length (cm)	61.72 ± 1.66 ^b^	65.91 ± 1.20 ^a^
Hip height (cm)	67.00 ± 1.79 ^a^	63.25 ± 1.29 ^b^
Shoulder height (cm)	64.01 ± 1.68 ^a^	62.38 ± 1.21 ^a^
Ear length (cm)	23.67 ± 0.88 ^a^	24.06 ± 0.63 ^a^
Ear width (cm)	8.91 ± 0.37 ^b^	9.93 ± 0.26 ^a^
Face length (cm)	19.95 ± 0.71 ^b^	20.82 ± 0.51 ^a^
Face width (cm)	9.72 ± 0.31 ^b^	10.53 ± 0.22 ^a^
Tail length (cm)	15.25 ± 0.64 ^b^	16.61 ± 0.46 ^a^
Withers width (cm)	25.40 ± 1.20 ^a^	25.02 ± 0.86 ^a^
Heart girth (cm)	69.31 ± 2.54 ^a^	71.18 ± 1.82 ^a^
Horn length (cm)	20.33 ± 1.44 ^a^	20.80 ± 1.00 ^a^
Tail circumference (cm)	2.90 ± 0.52 ^b^	4.51 ± 0.37 ^a^
Shoulder width (cm)	20.00 ± 0.93 ^a^	20.97 ± 0.67 ^a^
Testicular circumference (cm)	20.96 ± 1.78 ^a^	21.51 ± 1.25 ^a^

Different superscript letters (a, b) within the same row indicate statistically significant differences between Ardi Bahraini and Ardi Mu’atar goats at *p* < 0.05. Traits with sex-specific measurement, such as testicular circumference, were analyzed only in relevant animals. Body weight was not included as a covariate when analyzed as a dependent variable.

**Table 3:** Genome-wide significant and suggestive SNPs associated with phenotypic differentiation between Ardi Bahraini and Ardi Mu’atar goats.

Locus	Chromosome	Position	FDR	Bonferroni
snp45555-scaffold622-996214	13	82,031,445	6.26 × 10^−10^	6.26 × 10^−10^
snp32665-scaffold3753-271193	14	73,435,116	3.59 × 10^−7^	3.59 × 10^−7^
snp41814-scaffold544-72928	6	50,510,087	4.17 × 10^−6^	1.01 × 10^−5^
snp23327-scaffold2330-184378	29	45,098,688	1.01 × 10^−5^	NS

**Table 4:** Candidate genomic regions near associated SNPs identified in Bahraini Ardi goat lines.

Locus	Chromosome	Position	P-V-Bonferroni	Genes/Variants
snp45555-scaffold622-996214	13	82,031,445	6.26 × 10^−10^	Variation ID:rs268277393 Alleles: A/G Type: SNV Position:82,031,445 *TRNAC-GCA* gene, and *DOK5* gene
snp32665-scaffold3753-271193	14	73,435,116	3.59 × 10^−7^	ID: rs268264845 Alleles: G/A Allele Type: SNV Position:73,435,116 *TMEM71* gene previous to Position (intron region) Post The SNP PHF20L1
snp23327-scaffold2330-184378	29	45,098,688	1.01 × 10^−5^	ID: rs268255751 Alleles: G/A Allele Type: SNV Position: 45,098,688 Before SNP position: *LOC102180829* gene after the SNP position: *SPTBN2* gene

Note: Alleles (e.g., A, G) indicate the nucleotide variants observed at each single-nucleotide polymorphism (SNP) locus.

**Table 5:** Minor allele frequencies of candidate SNPs associated with phenotypic differentiation between Ardi Bahraini and Ardi Mu’atar goats.

CHR No	SNP Name	Ardi Line	Allele A1	Allele A2	MAF
13	snp45555-scaffold622-996214	Ardi Bahraini	A	G	0.5
13	snp45555-scaffold622-996214	Ardi Mu’atar	A	G	0.0556
6	snp41814-scaffold544-72928	Ardi Bahraini	A	G	0.325
6	snp41814-scaffold544-72928	Ardi Mu’atar	A	G	0.305
14	snp32665-scaffold3753-271193	Ardi Bahraini	G	A	0.4
14	snp32665-scaffold3753-271193	Ardi Mu’atar	G	A	0.152
29	snp23327-scaffold2330-184378	Ardi Bahraini	G	A	0.2875
29	snp23327-scaffold2330-184378	Ardi Mu’atar	G	A	0.444

Note: A1 and A2 represent the two alleles observed at each SNP locus; allele notation (e.g., A, G) refers to nucleotide variants. MAF = minor allele frequency.

**Table 6:** Allele and genotype frequencies of candidate SNPs associated with phenotypic differentiation between the two Bahraini Ardi goat lines.

Chr. No	Locus Name		Allele A1	Allele A2	Homozygous A1A1	Heterozygous A1A2	Homozygous A2A2
13	snp45555-scaffold622-996214		A	G			
		Ardi Bahraini	0.50	0.50	11	18	11
		Ardi Mu’atar	0.055	0.945	1	2	33
6	snp41814-scaffold544-72928		A	G			
		Ardi Bahraini	0.325	0.675	4	18	18
		Ardi Mu’atar	0.305	0.695	1	21	14
14	snp32665-scaffold3753-271193		G	A			
		Ardi Bahraini	0.40	0.60	7	18	15
		Ardi Mu’atar	0.152	0.848	2	7	27
29	snp23327-scaffold2330-184378		G	A			
		Ardi Bahraini	0.287	0.713	6	11	23
		Ardi Mu’atar	0.444	0.556	4	24	8

Note: A1 and A2 represent the two alleles at each SNP locus; allele notation (e.g., A, G) corresponds to nucleotide variants. Genotype classes (A1A1, A1A2, A2A2) indicate homozygous and heterozygous combinations.

## Data Availability

The datasets generated and analyzed during this study are subject to data governance arrangements under the FAO–Bahrain project (UTF/BAH/006/BAH), in which the Government of the Kingdom of Bahrain retains overall responsibility. Therefore, the data are not publicly available. Access may be granted by the corresponding author upon reasonable request and subject to authorization from the relevant national authorities.

## Abbreviations

The following abbreviations are used in this manuscript:

ANOVA	Analysis of variance
ASIP	Agouti signaling protein
bp	Base pair
Chr.	Chromosome
DOK5	Docking protein 5
DNA	Deoxyribonucleic acid
FAO	Food and Agriculture Organization of the United Nations
FDR	False discovery rate
FTA card	Flinders Technology Associates card
GLM	General linear model
GWAS	Genome-wide association study
HWE	Hardy–Weinberg equilibrium
LD	Linkage disequilibrium
MAF	Minor allele frequency
MAS	Marker-assisted selection
MC1R	Melanocortin 1 receptor
NCBI	National Center for Biotechnology Information
PCA	Principal component analysis
PHF20L1	PHD finger protein 20-like 1
PLINK	Whole-genome association analysis toolset
Q–Q plot	Quantile–quantile plot
QC	Quality control
RTM-GWAS	Restricted two-stage multi-locus multi-allele genome-wide association study
SAS	Statistical Analysis System
SNP	Single nucleotide polymorphism
SPTBN2	Spectrin beta, non-erythrocytic 2
TMEM71	Transmembrane protein 71
TRNAC-GCA	Transfer RNA cysteine, anticodon GCA
TYRP1	Tyrosinase-related protein 1
UTF/BAH/006/BAH	FAO–Bahrain project code for the trust fund project under which the study was conducted
